# EGL-13/SoxD Specifies Distinct O_2_ and CO_2_ Sensory Neuron Fates in *Caenorhabditis elegans*

**DOI:** 10.1371/journal.pgen.1003511

**Published:** 2013-05-09

**Authors:** Jakob Gramstrup Petersen, Teresa Rojo Romanos, Vaida Juozaityte, Alba Redo Riveiro, Ingrid Hums, Lisa Traunmüller, Manuel Zimmer, Roger Pocock

**Affiliations:** 1 Biotech Research and Innovation Centre, University of Copenhagen, Copenhagen, Denmark; 2 Research Institute of Molecular Pathology, Vienna, Austria; Harvard University, United States of America

## Abstract

Animals harbor specialized neuronal systems that are used for sensing and coordinating responses to changes in oxygen (O_2_) and carbon dioxide (CO_2_). In *Caenorhabditis elegans*, the O_2_/CO_2_ sensory system comprises functionally and morphologically distinct sensory neurons that mediate rapid behavioral responses to exquisite changes in O_2_ or CO_2_ levels via different sensory receptors. How the diversification of the O_2_- and CO_2_-sensing neurons is established is poorly understood. We show here that the molecular identity of both the BAG (O_2_/CO_2_-sensing) and the URX (O_2_-sensing) neurons is controlled by the phylogenetically conserved SoxD transcription factor homolog EGL-13. *egl-13* mutant animals fail to fully express the distinct terminal gene batteries of the BAG and URX neurons and, as such, are unable to mount behavioral responses to changes in O_2_ and CO_2_. We found that the expression of *egl-13* is regulated in the BAG and URX neurons by two conserved transcription factors—ETS-5(Ets factor) in the BAG neurons and AHR-1(bHLH factor) in the URX neurons. In addition, we found that EGL-13 acts in partially parallel pathways with both ETS-5 and AHR-1 to direct BAG and URX neuronal fate respectively. Finally, we found that EGL-13 is sufficient to induce O_2_- and CO_2_-sensing cell fates in some cellular contexts. Thus, the same core regulatory factor, *egl-13*, is required and sufficient to specify the distinct fates of O_2_- and CO_2_-sensing neurons in *C. elegans*. These findings extend our understanding of mechanisms of neuronal diversification and the regulation of molecular factors that may be conserved in higher organisms.

## Author Summary

During the development of an organism, certain neurons are programmed to perform specific tasks. For example, motor neurons coordinate locomotion and sensory neurons recognize specific environmental cues. The molecular mechanisms that generate specific neuronal classes are not fully understood. We investigated mechanisms that control the development of two distinct classes of neurons that are required for the nematode *Caenorhabditis elegans* to sense the respiratory gases O_2_ or CO_2_. In this study, we identified and characterized a conserved transcription factor, *egl-13*, that is required for the development of both of these classes of neurons. *egl-13* is related to the SoxD family of transcription factor proteins in vertebrates. We found that *egl-13* controls the production of specific proteins that provide these cells with the ability to sense both O_2_ and CO_2_. Further, we found that *egl-13* works in conjunction with two additional factors, *ahr-1* and *ets-5*, to regulate this developmental decision. This work provides new insight into how transcriptional regulatory networks specify different but related neuronal identities and provides a platform for future studies to understand how neuronal diversity is generated.

## Introduction

The capacity of the nervous system to sense and respond to fluctuations in the external and internal environment is essential for homeostasis and survival. Neuronally controlled homeostatic buffering is delivered through cellular and systemic physiological adjustments and by seeking optimal environmental conditions through behavioral strategies [1]–[4]. A crucial homeostatic capacity of animals is the ability to sense and respond to changes in concentration of the respiratory gases oxygen (O_2_) and carbon dioxide (CO_2_) [5], [6]. O_2_ is essential for the generation of energy in the form of adenosine triphosphate (ATP); however, O_2_ also exerts toxicity through the production of reactive oxygen species (ROS) [1]–[4], [7]. CO_2_ is a by-product of oxidative metabolism and prolonged exposure leads to acidosis [5], [6], [8]. CO_2_ is also an environmental cue used in host- and mate- finding and can initiate both aversive or attractive behaviors [9]–[11]. The evolution of mechanisms required to sense and respond to O_2_ and CO_2_ is therefore paramount for survival.

In *Drosophila*, specific sensory systems respond to external O_2_ levels [1], [7], [12]. In addition, *Drosophila* uses specialized olfactory and gustatory neurons to detect CO_2_ changes via specialized chemosensory receptors called Gr21a/Gr63a [9], [13]. In humans, O_2_, CO_2_ and pH levels are monitored by specific regions of the brainstem and by specialized neurosecretory glomus cells of the carotid body [14], whereas in non-human mammals CO_2_ is also sensed by specific olfactory neurons that target the necklace glomeruli in the olfactory bulb via the guanylyl cyclase GC-D [15]. It is poorly understood how the specification of such specialized sensory neurons is regulated. However, recent work in *Drosophila* has shown that epigenetic mechanisms play an important role [16].

Respiratory gas sensing is a crucial modality for *Caenorhabditis elegans* whose natural environment, such as rotting fruit and compost, can have wide ranges of O_2_ and CO_2_ levels [17]. Previous work has shown that in the laboratory, worms have a behavioral preference for 5%–10% O_2_ and are exquisitely sensitive to minor changes in O_2_ concentration [18], [19]. In addition, worms mount avoidance responses to CO_2_ levels above 0.5% [4], [11]. Of the 302 neurons in the C. *elegans* nervous system, at least six neurons are specifically dedicated to the detection and response to changes in O_2_ and CO_2_ levels. These include the BAGL/R, URXL/R, AQR and PQR neurons. The BAG neurons are the primary CO_2_ sensors and they also respond to decrease in O_2_ concentration [11], [20]–[22]. The URX, AQR and PQR neurons are specialized for responding to increasing O_2_ concentrations [20]. In *C. elegans*, members of the guanylyl cyclase family of proteins are crucial factors required for O_2_ and CO_2_ sensing. Pioneering work revealed that the soluble guanylyl cyclases (sGCs) GCY-35 and GCY-36 mediate high O_2_ avoidance behavior via the URX, AQR and PQR neurons and that GCY-35 directly binds to molecular O_2_ [18]. In contrast, the sGCs, GCY-31 and GCY-33 function in the BAG neurons to sense decreases in O_2_ [20]. Recent work found that the membrane-bound receptor-type guanylyl cyclase GCY-9 acts specifically in the BAG neurons to mediate CO_2_ avoidance behavior [21]. Other molecules such as the Phe-Met-Arg-Phe-NH_2_ (FMRF-amide)-related peptides (FLP-8, FLP-13, FLP-17 and FLP-19) are expressed in either a subset or all of the O_2_- and CO_2_-sensing neurons; however their precise molecular functions in O_2_ and CO_2_ sensing are not known [23], [24].

Neuronal specialization within the O_2_/CO_2_-sensing system in *C. elegans* is an excellent model to study the control of neuron diversity. The O_2_-sensing (URX, AQR and PQR) and O_2_/CO_2_-sensing (BAG) neurons have overlapping and non-overlapping patterns of guanylyl cyclase and neuropeptide expression, which are reflected in their related, albeit distinct functionalities [20]. At present, it is unclear how the expression of these molecules is restricted to certain parts of the O_2_/CO_2_-sensing nervous system, and how such restrictions coordinate neuronal fate and function.

Here, we have identified the Sox transcription factor EGL-13 as an important regulator of the O_2_ and CO_2_-sensing neuron cell fate decision. EGL-13 is required for the expression of distinct proteins required for sensing both O_2_ and CO_2_ and as such, *egl-13* mutant animals are unable to mount behavioral responses to changes in O_2_ and CO_2_. We found that the expression of EGL-13 is controlled by ETS-5 in the BAG neurons and by AHR-1 in the URX neurons, and acts in partially parallel pathways with these factors to drive neuronal fate. Finally, we found that EGL-13 is sufficient to drive O_2_- and CO_2_-sensing cell fates in certain cellular contexts. Therefore, EGL-13 is a core regulatory factor that is both required and sufficient to drive O_2_- and CO_2_-sensing neuron specification in *C. elegans*. As EGL-13 is a member of the SoxD family of transcription factors, we anticipate that the regulatory relationships described here will provide a paradigm for the control of neuronal fate specification by Sox proteins in other cellular contexts.

## Results/Discussion

### The SoxD transcription factor *egl-13* specifies distinct O_2_- and CO_2_-sensing neurons

**Figure 1:**
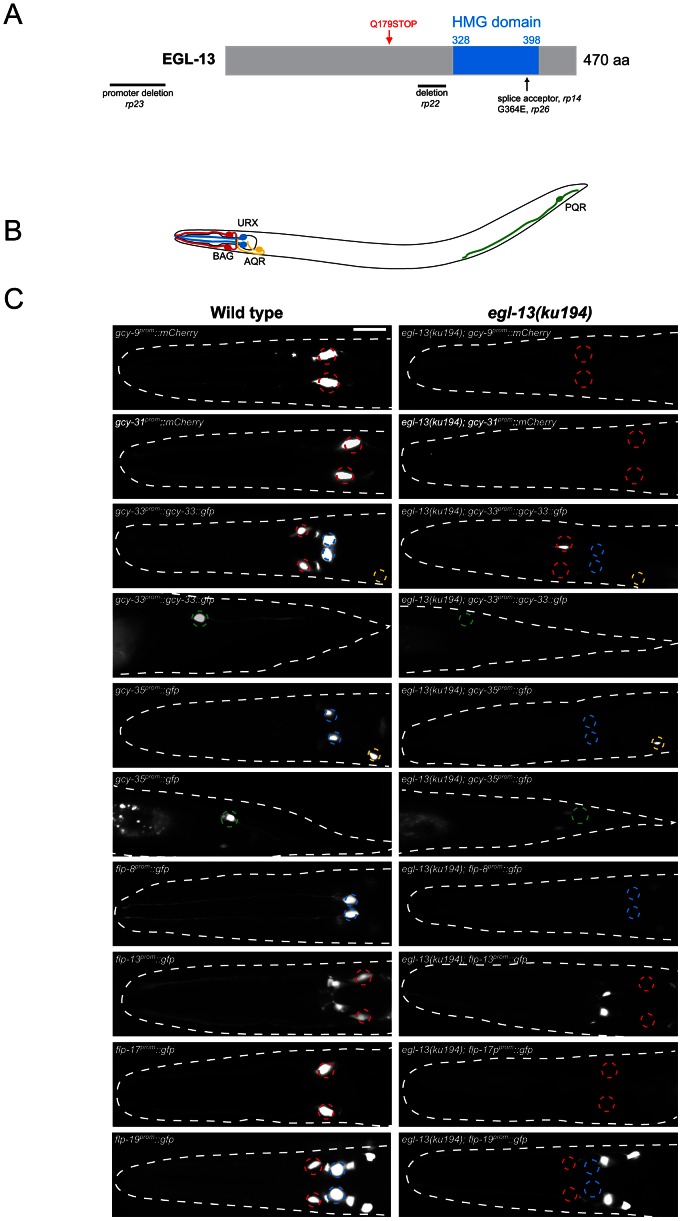
*egl-13* is required for O_2_- and CO_2_-sensing neuron specification. (A) Molecular identity of mutant alleles obtained from the forward genetic screens. *egl-13* alleles first described in this article are shown in black (*rp14*, *22*, *23*, and *26*) and the previously described *ku194* [25] is shown in red. The nature of the molecular lesions is as follows: *rp14* is a splice acceptor mutation between exons 8 and 9, causing a premature termination codon within the HMG domain. *rp22* is an out-of-frame 317 bp deletion between exons 6 and 7 that leads to a predicted premature STOP codon in the EGL-13 protein that lacks the HMG domain. The *rp23* allele is a promoter deletion, removing 1128 bp between −1700 and −572 upstream of the ATG codon. *rp26* is a G-to-A transition that converts a highly conserved glycine to a glutamic acid in the HMG domain. The isolated *egl-13* mutants are therefore predicted to either abrogate DNA binding (*rp14*, *rp22* and *rp26*) or reduce/eliminate expression of *egl-13* transcript (*rp23*). (B) Schematic of the O_2_- and CO_2_-sensing system. Anterior to the left. (C) Fluorescence reporter expression of the O_2_- and CO_2_-sensing neuron terminal gene batteries in wild-type (left) and *egl-13(ku194*) mutant (right) at the young adult stage. Quantification of data and information on reporter strains is shown in Table S1. Neuron positions are marked with dashed circles: BAG(red), URX(blue), AQR(yellow) and PQR(green). Fluorescent cells not marked with circles are non-O_2_/CO_2_-sensing neurons in the respective strains and their expression is unaffected by loss of *egl-13*. Scale bar, 20 µm. Anterior to the left.

In order to identify molecules and pathways important for O_2_- and CO_2_-sensing neuron specification, we have taken a forward genetics approach in *C. elegans*. We isolated four independent allelic mutations (*rp14*, *rp22*, *rp23* and *rp26*) that affect the expression of terminal differentiation markers in the O_2_ and/or CO_2_-sensing neurons (Figure 1 and Table S1) Mutant hermaphrodites of each of these alleles are severely egg-laying defective (Egl) and form a bag-of-worms where embryos hatch inside the mother (Figure S1). We investigated their vulval phenotype and found that the anchor cell fails to fuse with the uterine seam cell, causing a blockage of the uterus and the resultant Egl phenotype (Figure S1). This anchor cell fusion defect is reminiscent of that observed in *egl-13(ku194*) mutant animals [25] which we found to also exhibit defects in O_2_ and CO_2_ reporter expression (Figure 1 and Figure S1). Subsequent Sanger sequencing of *rp14*, *rp22*, *rp23* and *rp26* revealed genetic lesions in the *egl-13* locus (Figure 1A). *egl-13* encodes the *C. elegans* ortholog of the HMG-domain-containing SoxD family of transcription factors that has no previously reported role in the worm nervous system.

### Loss of *egl-13* affects terminal fate of O_2_- and CO_2_-sensing neurons

The BAG, URX, AQR and PQR neurons in *C. elegans* are required for sensing and responding to fluctuations of O_2_ and CO_2_ levels in the environment [11], [18], [19]. Distinct batteries of genes are expressed in these neurons that are predicted to provide the optimal functionality required for O_2_ and CO_2_ sensing, however the role of only a subset of these genes has been analyzed in detail [20], [21], [26]. We used fluorescent reporter constructs to monitor expression of these gene batteries to understand how *egl-13* controls O_2_ and CO_2_-sensing neuron cell fate (Figure 1). We analyzed the expression of guanylyl cyclases (*gcy-9*, *gcy-31*, *gcy-33*, *gcy-35* and *gcy-36*) and Phe-Met-Arg-Phe-NH_2_ (FMRF-amide)-related peptides (*flp-8*, *flp-13*, *flp-17* and *flp-19*) that are all terminal differentiation genes expressed in all or a subset of O_2_ and CO_2_-sensing neurons [18], [19], [21], [24]. We crossed these reporter transgenes into *egl-13* mutant animals (*ku194* allele) and found that none of the reporters were properly expressed in *egl-13* mutants (Figure 1 and Table S1). We also found similar effects in the four *egl-13* mutant alleles we isolated (*rp14*, *rp22*, *rp23* and *rp26*) (Figure S1C). We noticed that some of the reporters were exquisitely sensitive to *egl-13* loss whereas others exhibited partially penetrant defects (Table S1). This suggests that the expression of some terminal differentiation factors are under the collaborative control of additional factors that are able to compensate for the loss of *egl-13*.

The BAG and URX neurons are derived from the AB lineage and are posterior sisters of other neurons that have distinct fates [27] (Figure S2). We therefore asked whether *egl-13* is also required for the specification of the sister cells of BAG or URX. We crossed *egl-13(ku194)* mutant animals into fluorescent reporter strains for the SMDV, *zfIs2 (lgc-55::mCherry)* and CEPD, *vtIs1* (*dat-1::gfp*), sister cells for BAG and URX neurons respectively. We found that the expression of these reporters were unaffected by loss of *egl-13* suggesting a specific role for *egl-13* in the posterior branch of these lineages (Table S1 and Figure S2). Taken together, we conclude that *egl-13* controls the expression of the distinct O_2_- and CO_2_-sensing neuron terminal gene batteries that distinguish them from lineage-related neurons.

### *egl-13* acts cell-autonomously in O_2_- and CO_2_-sensing neurons

**Figure 2:**
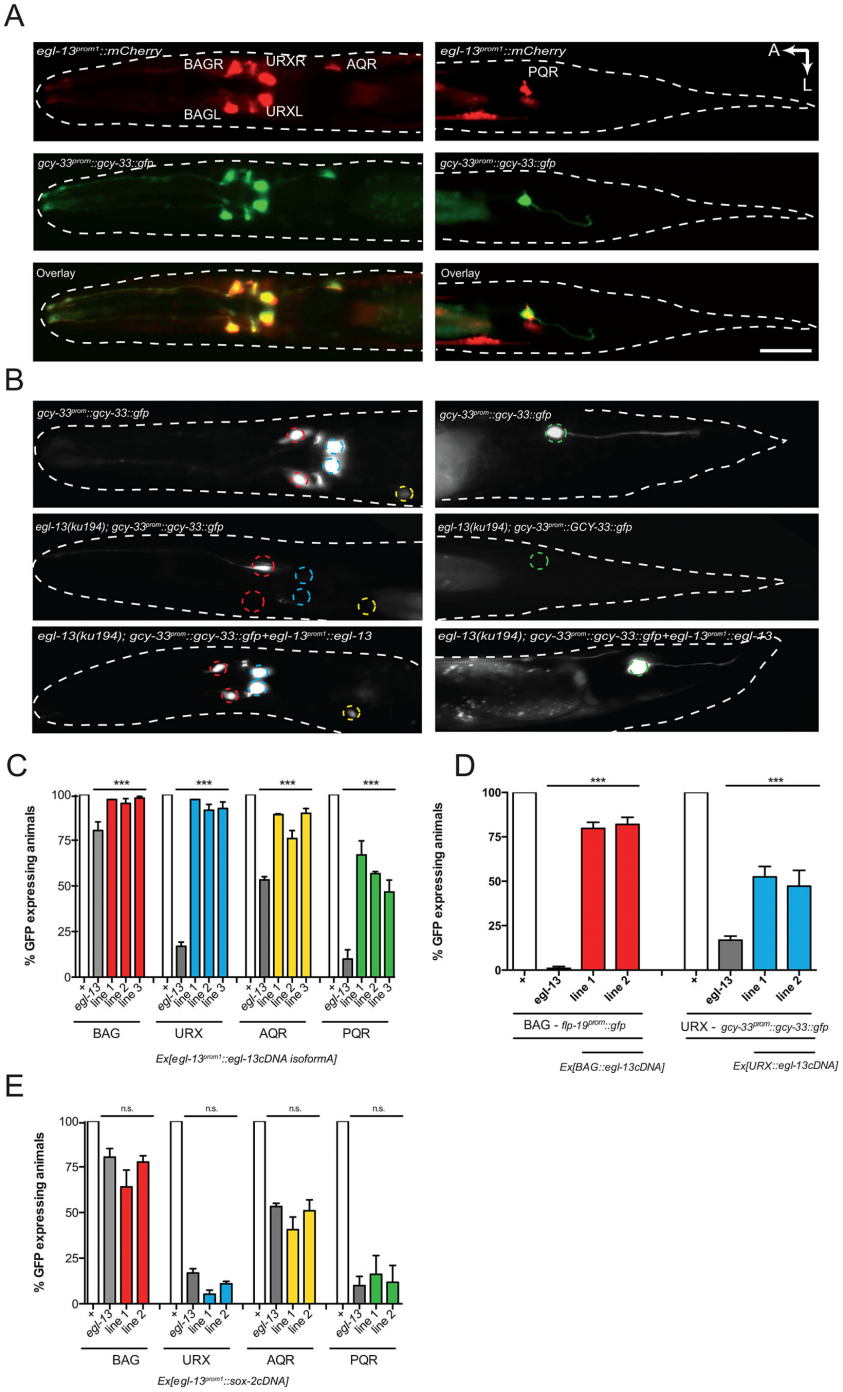
*egl-13* functions cell autonomously to drive O_2_- and CO_2_-sensing neuron cell fate. (A) Dorsal view of a young adult hermaphrodite expressing an *egl-13^prom1^::mCherry* transcriptional reporter transgene (top panels), a *gcy-33^prom^::gcy-33::gfp* translational reporter marking BAGL/R, URXL/R, AQR and PQR (center panels) and a merge of the two pictures showing co-localization in the O_2_- and CO_2_-sensing neurons (bottom panels). Left hand panels show the head region and right hand panels the tail region. We also observed expression of *egl-13^prom1^::mCherry* in muscle and vulval cells (not shown). A: anterior, L: left. The scale bar in lower panel is 20 µm. The *egl-13^prom1^::mCherry* transgene is *rpEx272* and the *gcy-33^prom^::GCY-33::gfp* transgene is *rpIs7*. (B) Fluorescence micrographs of *gcy-33^prom^::gcy-33::gfp* expression in a wild-type animal (top panels), an *egl-13(ku194)* mutant animal (center panels) and an *egl-13(ku194)* mutant animal rescued by transgenic expression of *egl-13^isoformA^cDNA* driven by the endogenous 3.5 kb *egl-13^prom1^* (bottom panels). Left hand panels show the head region and right hand panels the tail region. (C) Transgenic expression of *egl-13^isoformA^cDNA* under the control of *egl-13^prom1^* rescues *egl-13* mutant neuronal phenotypes, in the *gcy-33^prom^::gcy-33::gfp* strain. n = 52–55. ***P<0.001. Lines 1–3 = independent transgenic rescue lines. See materials and methods for neuronal scoring criteria used. (D) Transgenic expression of *egl-13^isoformA^cDNA* under the control of the *gcy-33^1kb^* (BAG-specific) or *unc-86^(700bp)^* (URX-specific) promoters rescues *egl-13* mutant neuronal phenotypes in the *flp-19^prom^::gfp* (BAG) and *gcy-33^prom^::gcy-33::gfp* (URX) strains. n = 39–55. ***P<0.001. Lines 1–2 = independent transgenic rescue lines. See materials and methods for neuronal scoring criteria used. (E) Transgenic expression of *sox-2* cDNA under the control of *egl-13^prom1^* is unable to rescue *egl-13* mutant neuronal phenotypes, in the *gcy-33^prom^::gcy-33::gfp* strain. n = 56–60. (n.s.) indicates no significant difference from non transgenic *egl-13* mutant animals. Lines 1–2 = independent transgenic rescue lines. See materials and methods for neuronal scoring criteria used.

To monitor *egl-13* expression, we generated two promoter-driven fluorescent reporters (*egl-13^prom1^::mCherry* and *egl-13^prom1^::gfp*) that contain 3.5 kb of *egl-13* upstream sequence (Figure 2A and Figure S3). Expression is first detected in 4 neuronal cells at around 350 min post-fertilization, which is the time at which the BAG and URX neurons are born (Figure S3). Expression is restricted to these 4 neurons during embryogenesis (Figure S3). At the first larval stage, *egl-13* expression is observed in the BAG and URX neurons plus occasionally in a small number of unidentified cells in the head and tail (including the AQR and PQR neurons) (Figure S3). Later during larval development, *egl-13* expression is observed in body wall muscle and vulval cells (data not shown). Neuronal expression is restricted to the O_2_ and CO_2_-sensing neurons in the adult (Figure 2A). Using the 3.5 kb *egl-13* promoter (*egl-13^prom1^*) we transgenically expressed *egl-13^isoformA^*cDNA in *egl-13(ku194)* mutant animals and were able to rescue both the defect in O_2_ and CO_2_-sensing neuron fate marker expression and the Egl phenotype (Figure 2B–2C, Figure S4 and data not shown). To confirm that *egl-13* acts cell autonomously to control O_2_ and CO_2_-sensing neuron fate, we used neuron-specific promoters to drive *egl-13^isoformA^*cDNA expression in the BAG or URX neurons (Figure 2D). We found that indeed neuron-specific expression of *egl-13* rescued the O_2_ and CO_2_-sensing neuron fate defect of *egl-13(ku194)* mutant animals (Figure 2D). Therefore, we conclude that *egl-13* acts autonomously in the BAG and URX neurons to direct their fate.

The *egl-13* gene has 4 predicted isoforms, all of which contain the same HMG DNA/protein binding domain, however they each have varying lengths of amino terminal tail. Such tails in SoxD proteins can cooperate with other factors to control gene expression [28]. We therefore tested whether the long N-terminal region of EGL-13^isoformA^ is required for its rescuing ability. We used *egl-13^prom1^* to drive EGL-13^isoformD^ (lacking 157 amino acids of the N-terminal tail of isoformA) in *egl-13(ku194)* animals and found that it fully rescued the defect in O_2_ and CO_2_-sensing neuron fate marker expression and the Egl phenotype (Figure S4 and data not shown). Thus, the EGL-13 N-terminal region is not required for its roles in vulval cell nor O_2_ and CO_2_-sensing neuron specification. We next asked whether SoxD proteins play specific roles in these decisions by attempting to rescue the *egl-13* mutant defects with the SoxB family member, *sox-2*. We expressed *sox-2* cDNA under the control of *egl-13^prom1^* in *egl-13(ku194)* mutant animals. We found that *sox-2* is unable to rescue O_2_ and CO_2_-sensing neuron fate marker expression (Figure 2E). These data indicate that the SoxD HMG domain plays a specific role in the specification of O_2_ and CO_2_-sensing neuron fate in *C. elegans*.

### *egl-13* is required and sufficient to induce O_2_-sensing neuron fate

**Figure 3:**
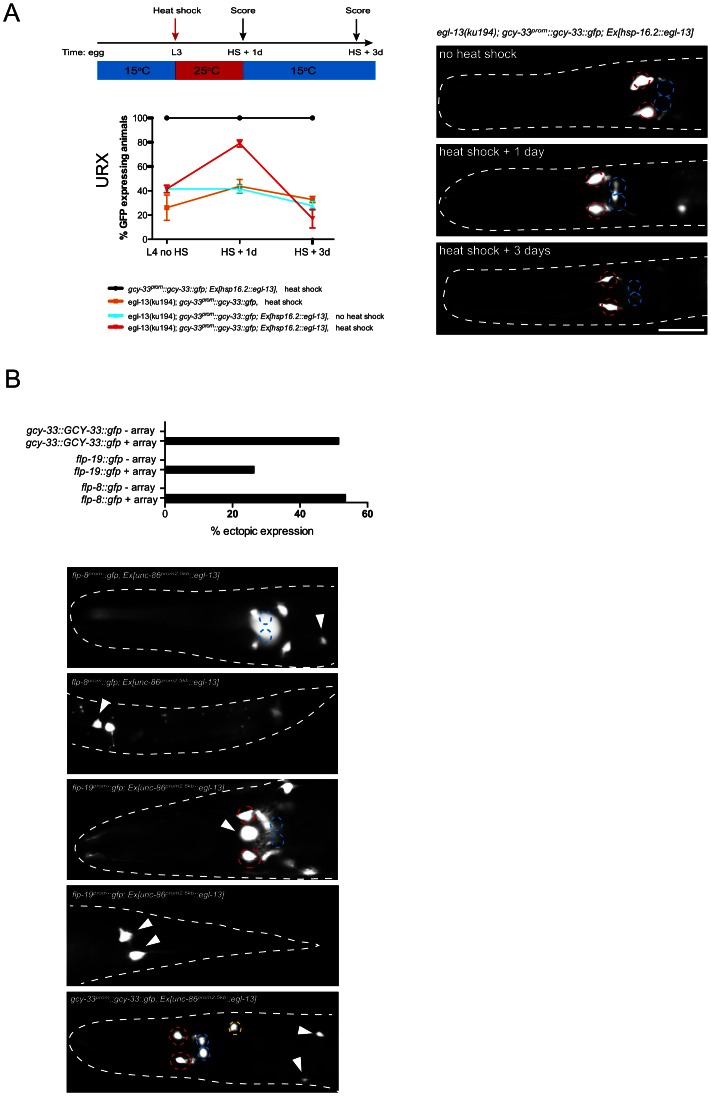
*egl-13* is required and sufficient to induce O_2_- and CO_2_-sensing neuron fate. (A) *egl-13* is required to maintain O_2_/CO_2_-sensing neuron fate. Induction of *egl-13* expression via transient heat-shock at the L2/3 stage restores *gcy-33^prom^::gcy-33::gfp* expression in the URX neurons of *egl-13(ku194)* mutant animals. A schematic describing the heat-shock protocol is shown at the top left. The number of positive neurons was assessed one day after heat-shock (L2/3+1 day). No increase in the number of positive neurons was observed in *egl-13(ku194); gcy-33^prom^::gcy-33::gfp*; Ex*[hsp-16.2::egl-13]* animals in the absence of heat shock. A significant decrease in the number of positive neurons was observed in *egl-13(ku194); gcy-33^prom^::gcy-33::gfp*; Ex*[hsp-16.2::egl-13]* animals 3 days after heat shock with growth at 15°C (L4+3 days) when compared to one day after heat shock (L4+1 day). Quantification of the number of *gcy-33^prom^::gcy-33::gfp* positive neurons is shown at the indicated time points. Error bars represent the standard error of the mean (SEM). Statistical model applied is a one-way ANOVA with Newman-Keuls multiple comparison test **P<0.05, ***P<0.005. Representative fluorescent micrographs on the right indicate the continual requirement for *egl-13* to maintain O_2_/CO_2_-sensing neuron fate. Neuron positions are marked with dashed circles: BAG(red), URX(blue). Two different extrachromosomal lines were analyzed for this experiment and showed similar effects (only one is shown). 53–113 animals were used per time point per genotype. See materials and methods section for heat shock protocol and for neuronal scoring criteria used. Scale bar, 20 µm. Anterior to the left. (B) *egl-13* is sufficient to induce O_2_/CO_2_-sensing cell fate markers. An *unc-86* promoter was used to drive broad neuronal expression of *egl-13* cDNA in a wild-type background. Graph indicates the percentage of animals that exhibit ectopic expression O_2_/CO_2_-sensing cell fate marker (+ array = transgenic animals expressing *unc-86^prom^::egl-13cDNA* array; − array = transgenic siblings not expressing the array). Representative fluorescent micrographs of ectopic expression of three O_2_/CO_2_-sensing cell fate markers: *flp-8^prom^::gfp*, *flp-19^prom^::gfp* and *gcy-33^prom^::gcy-33::gfp*. Neuron positions are marked with dashed circles: BAG(red), URX(blue) and ectopic expression of each reporter is marked with arrowheads. Scale bar, 20 µm. Anterior to the left.

We have shown that *egl-13* is expressed throughout the life of the worm in the O_2_ and CO_2_-sensing neurons; and is required to induce terminal differentiation features. To ask whether *egl-13* is required continuously to maintain the expression of the terminal gene battery of these neurons, we sought to postdevelopmentally remove *egl-13* gene activity. *egl-13* gene activity could not be removed by RNA-mediated interference in an RNAi sensitized background (data not shown) and there are no temperature-sensitive alleles of *egl-13* available. Instead, we generated animals that lack endogenous EGL-13 protein but express heat-shock inducible *egl-13* cDNA from an extrachromosomal array under the control of the *hsp-16.2* promoter (Figure 3). We focused our analysis on the URX neurons and found that the loss of *gcy-33^prom^::gcy-33::gfp* reporter expression in *egl-13(ku194)* worms could be rescued through heat-shock induction of *egl-13* during mid-larval stages (Figure 3A). This indicates that O_2_-sensing neurons generated during embryogenesis persist in an *egl-13*-responsive state. These neurons are, therefore, not converted into another fate when *egl-13* is lost; however, they do not acquire the terminal O_2_-sensing neuron differentiation program. When *egl-13* activity was supplied transiently, through removal of heat-shock stimulus, we observed a gradual loss of reporter expression during adulthood in the URX neurons (Figure 3A). Therefore, *egl-13* gene activity is continuously required to maintain URX cell fate. To ask whether misexpression of *egl-13* in other neurons is sufficient to induce O_2_ and CO_2_ terminal fate we expressed *egl-13* under the control of an early neuronal promoter (Figure 3B). We found that *egl-13* is indeed sufficient to induce expression of O_2_ and CO_2_ terminal fate markers in some cellular contexts (Figure 3B). This suggests that *egl-13* is not only required but also sufficient to induce O_2_ and CO_2_-sensing neuron fate in specific contexts, which is similar to previous studies of terminal selector genes [29]–[31]. The restricted induction we observed may be dependent on the embryonic time-point of induction or the expression of other unknown co-factors that are required for induction of O_2_ and CO_2_-sensing neuron fate.

### *egl-13* mutants are defective in O_2_ and CO_2_ sensing

**Figure 4:**
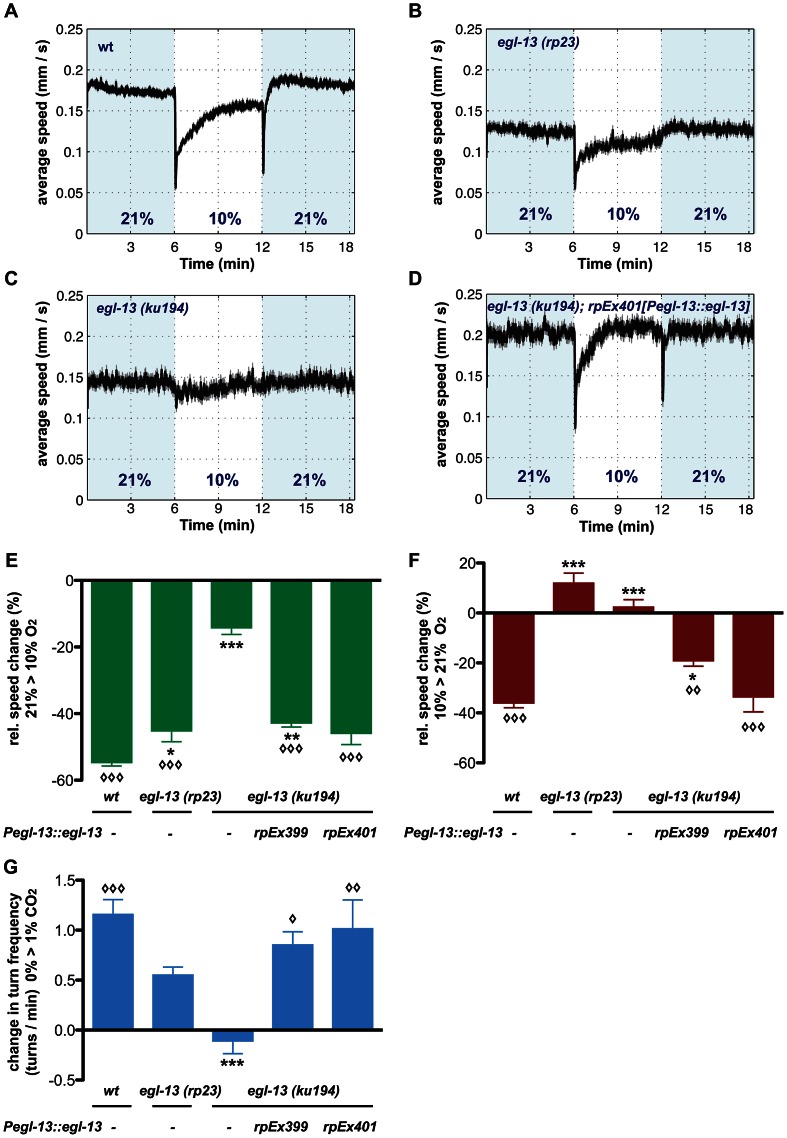
*egl-13* is essential for locomotion responses to O_2_ and CO_2_ concentration shifts. (A-D) Locomotion speed of *C. elegans* during O_2_ concentration shifts. Traces show average forward speed and dark shading indicates standard error of the mean (SEM). O_2_ concentrations were switched between 21% and 10%. Shading represents intervals at 21%. (A) Wild-type animals. (B) *egl-13(rp23)* mutants. (C) *egl-13(ku194)* mutants. (D) Rescue of *egl-13(ku194)* mutant phenotype by transgenic expression of *egl-13* cDNA under control of its own promoter. Transgene *rpEx401*. Note the respective reduction of speed levels in N2 after up- and downshift, which are abolished in *egl-13(ku194)* and restored in the transgenic line. *egl-13(rp23)* animals are affected mostly in their response to O_2_ upshift. (E, F) Quantification of data in A–D. Average speed changes in percent from basal speed to O_2_ downshift (E) and upshift (F) of animals with indicated genotypes. Transgenic rescue lines are significantly different from *egl-13(ku194)* mutant animals. (G) Average changes in omega turn frequency of animals with indicated genotypes, in response to 1% CO_2_. The defect in omega turn responses seen in *egl-13(ku194)* animals is restored in the transgenic lines (*rpEx399* and *rpEx401*). *egl-13(rp23)* animals only exhibit a partial defect. Error bars = SEM. Symbols indicate all significant differences one-way ANOVA with Bonferroni's Multiple Comparison Test (*/◊ p = 0.01–0.05, **/◊◊ p = 0.001–0.01, ***/◊◊◊ p<0.001). Asterisks indicate significant difference compared to wild-type, while diamonds indicate significant difference compared to *egl-13(ku194)* mutants. Data were calculated from n = 3 independent experiments for each mutant and transgenic rescue strain, and n = 6 independent experiments for wild-type. Each individual experiment was performed on 60–70 animals.

The crucial role for *egl-13* in O_2_ and CO_2_-sensing neuron fate determination suggested that *egl-13* mutant animals would be defective in O_2_ and CO_2_ sensing. We applied three behavioral paradigms that have been previously reported to be specific to either one of these neuron classes: BAG neurons modulate the animals' locomotion speed in response to an oxygen downshift from 21% O_2_ towards 10% O_2_ (Figure 4A, 4E) [20]. In addition, BAG neurons detect increases in CO_2_ concentrations, which trigger reorientation movements (omega turns) (Figure 4G) [11], [21]. URX neurons modulate the animals' locomotion speed in response to O_2_ upshifts towards 21% O_2_ (Figure 4A, 4F) [20]. We applied these behavioral assays to test how BAG and URX neurons are functionally affected in *egl-13* mutants. We tracked animals in a chamber without food, in an air-flow that switched between 21% O_2_ and 10% O_2_, or between 0% CO_2_ and 1% CO_2_. In contrast to wild-type animals, *egl-13(ku194)* mutant animals do not slow their locomotion in response to O_2_ upshift or downshift (Figure 4A, 4C, 4E, 4F). We found that *egl-13(ku194)* mutants are also defective in CO_2_ sensing since they fail to slow or perform omega turns in response to CO_2_ (Figure 4G). O_2_ and CO_2_ behavioral defects of *egl-13(ku194)* mutants are fully rescued when *egl-13* cDNA is resupplied under the control of *egl-13^prom1^* (Figure 4D–4G). These data confirm that *egl-13* is crucial for the specification and function of O_2_ and CO_2_-sensing system in *C. elegans*.

### The *egl-13* promoter contains neuron-specific regulatory modules

**Figure 5:**
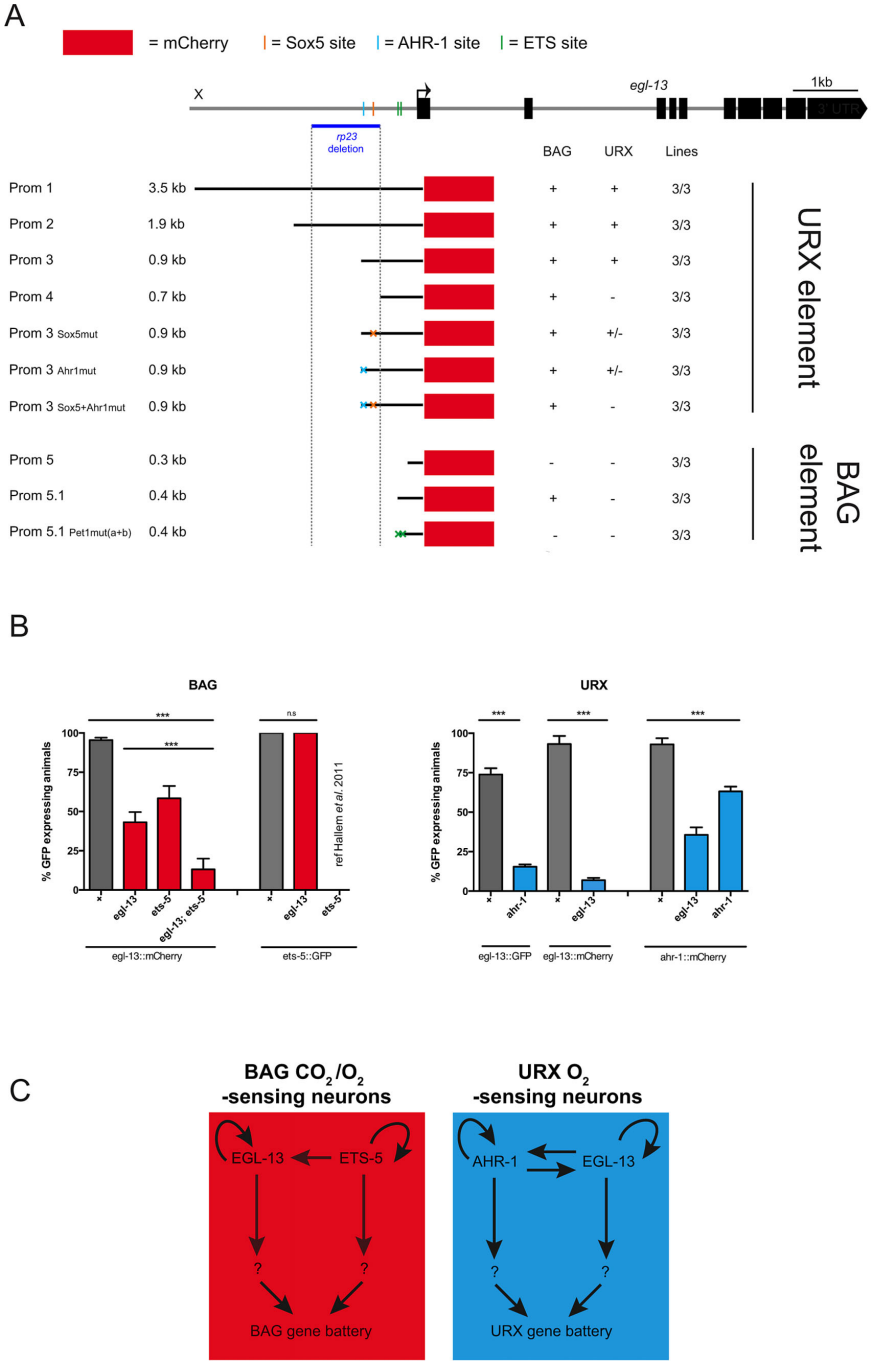
Independent regulatory modules drive *egl-13* expression in O_2_ versus O_2_/CO_2_-sensing neurons. (A) *egl-13* promoter analysis. Schematic representation of the *egl-13* locus with its 3.5 kb upstream region. The ATG codon is marked with an arrow and the exons are represented as black blocks. The upstream region deleted in the *rp23* allele is indicated with a blue horizontal line and a grey dashed vertical line. Below is a representation of cloned and injected constructs, and their expression pattern in the URX and BAG neurons. Black lines denote the promoter fragment placed in front of mCherry fluorescent protein (red boxes). Orange, blue and green crosses represent mutated Sox5, AHR-1 and ETS binding sites respectively. “+” indicates consistent reporter expression in at least 50% of animals in all lines. “+/−” indicates less than 50% of animals expressing the reporter in all lines. “−” indicates loss of expression. Three independent transgenic lines were analyzed for each promoter as indicated. (B) Expression of *egl-13* and *ets-5* reporter transgenes in the BAG neurons in the reciprocal mutant backgrounds (left). *egl-13(ku194)* and *ets-5(tm1734)* alleles were used. Expression of *egl-13* and *ahr-1* reporter transgenes in the URX neurons in the reciprocal mutant backgrounds (right). *egl-13(ku194)* and *ahr-1(ia3)* alleles were used. n>50 per strain. Error bars represent the standard error of the mean (SEM) **P<0.05, ***P<0.005. (n.s.) indicates no significant difference from wild-type. See materials and methods for neuronal scoring criteria used. (C) In the BAG neurons (left), the ETS transcription factor, ETS-5 regulates the expression of *egl-13* in the BAG neurons via conserved ETS binding sites located in the *egl-13* promoter. In addition, *egl-13* and *ets-5* autoregulate in the BAG neurons to generate BAG fate. In the URX neurons (right), *egl-13* and *ahr-1* autoregulate, in addition to regulating the expression of each other. Other unknown factors act in parallel to and in combination with these factors to drive BAG and URX neuronal fate.

One of the *egl-13* mutant alleles retrieved from our screen was a promoter deletion mutant (*rp23*). The *rp23* deletion removes 1128 bp of *egl-13* promoter from −1700 to −572 upstream of the translational start site (Figure 1A and Figure 5A). Intriguingly, the *rp23* mutation affects terminal marker expression in the URX but not BAG sensory neurons and is mostly defective in URX and less affected in BAG regulated behaviors (Figure 4B, 4E–4G; Figure S4C; and Table S1). This suggests that the *rp23* promoter deletion removes element(s) required to drive *egl-13* in the URX neurons while leaving the BAG-specific element(s) intact. To identify which upstream factors drive expression of *egl-13* in the molecularly and functionally distinct BAG and URX neurons we performed promoter deletion analysis, using the 3.5 kb upstream element (*egl-13^prom1^*) as a template. We generated transgenic worms expressing truncated versions of *egl-13^prom1^* driving *mCherry* or *gfp* protein and focused our expression analysis on BAG and URX regulation (Figure 5A). A 900 bp fragment (*egl-13^prom3^*), which includes 360 bp corresponding to the 3′ end of the *rp23* deletion, drove expression in BAG and URX. However, a 691 bp fragment (*egl-13^prom4^*), which lacks the missing region in the *rp23* deletion, only drove expression in the BAG neurons. Therefore, an important element required for *egl-13* expression specifically in the URX neurons lies within the 200 bp region included in *egl-13^prom3^*. Bioinformatic analysis of this region revealed that there are two conserved motifs that are potential binding sites for EGL-13/SOX5 itself and AHR-1, an aryl hydrocarbon receptor bHLH protein. Interestingly, *ahr-1* was previously shown to be required for the expression of some URX terminal fate markers [32]. Site-directed mutagenesis of the predicted AHR-1 binding site significantly reduced *egl-13^prom1^::mCherry* expression and a subsequent mutation in the putative EGL-13 binding site further reduced expression (Figure 5A). This suggests that both AHR-1 and EGL-13 regulate egl-13 expression. To test this, we crossed *egl-13^prom1^mCherry/gfp*-expressing animals into *egl-13(ku194)* and *ahr-1(ia3)* mutants and found that URX expression was reduced in both cases (Figure 5B). Therefore, AHR-1 and EGL-13 both contribute to the control of *egl-13* expression in the URX neurons.

To identify the regulatory module(s) that control *egl-13* expression in the BAG neurons, we continued to dissect the *egl-13* promoter. We identified a 432 bp region (*egl-13^prom5.1^*), immediately upstream of the ATG codon, which is sufficient to drive expression in the BAG neurons. Intriguingly, we found two conserved ETS-5/Pet1 binding sites in this region (Figure 5). Previous work identified ETS-5 as a crucial factor required for the specification of the BAG neurons, suggesting that ETS-5 may regulate *egl-13* expression in these neurons [26], [33]. We used site-directed mutagenesis to eliminate the ETS-5/Pet1 binding sites individually and in combination, and found that when both ETS-5/Pet1 binding sites are mutated the expression of *egl-13* is abrogated in the BAG neurons (Figure 5A). This suggests that ETS-5 directly regulates the expression of *egl-13* in the BAG neurons via conserved binding sites. We crossed the *ets-5(tm1734)* mutant into the *egl-13^prom1^::mCherry* strain and indeed found that BAG expression was affected (Figure 5B). In addition, we found that *egl-13* can regulate its own expression in the BAG neurons independently of *ets-5* via an, as yet, unidentified mechanism (Figure 5B).

Taken together, these data indicate that control of *egl-13* expression is coordinated by two independent regulatory mechanisms. In the O_2_-sensing URX neurons, *egl-13* expression is predominantly regulated by AHR-1 (Figure 5C). In contrast, an independent promoter module controlled by ETS-5 regulates *egl-13* expression in the O_2_/CO_2_-sensing BAG neurons (Figure 5C). In addition, *egl-13* is able to autoregulate in both the URX and BAG neurons (Figure 5C).

### *egl-13* acts in partially parallel pathways with *ets-5* and *ahr-1*

Previous work has identified *ets-5* and *ahr-1* as regulators of BAG and URX specification respectively [26], [32], [33] and we have shown that these factors are predominantly required to drive *egl-13* expression in these cells. To understand how these factors function together to coordinate BAG and URX specification, we analyzed the expression of terminal fate markers in single and double mutant combinations, where appropriate. We analyzed three URX markers (*flp-8::gfp*, *flp-19::gfp* and *gcy-33^prom^::gcy-33::gfp*) and six BAG markers (*flp-13::gfp*, *flp-17::gfp*, *flp-19::gfp*, *gcy-9::gfp*, *gcy-31::gfp* and *gcy-33^prom^::gcy-33::gfp*) and compared the effect of the individual loss of *egl-13*, *ets-5* and *ahr-1* (Figure S5).

The first observation from this analysis was that the expression of a subset of terminal differentiation markers is completely dependent on *egl-13* and one of the other factors acting in a linear pathway. For example, we find that BAG expression of *flp-13::gfp* and *flp-19::gfp* is almost 100% affected in both the *egl-13* and *ets-5* single mutants (Figure S5). This suggests that for these markers *egl-13* and *ets-5* act in the same pathway to drive marker expression. In contrast, expression of *gcy-9::mCherry* is completely dependent on *ets-5* with *egl-13* playing a minor role in its regulation (Figure S5). At the other end of the spectrum, *ets-5* and *egl-13* are minimally required to drive *gcy-31::mCherry* expression in the BAG neurons suggesting other factor(s) control the expression of this terminal fate marker (Figure S5). Taken together, these data indicate that *egl-13* and *ets-5* act in partially parallel pathways to drive BAG cell fate and that other unknown factors possibly act in a combinatorial manner to drive specific aspects of BAG fate.

We also observed differential effects of *egl-13* loss with URX terminal fate markers. Expression of the *flp-8::gfp* reporter is partially affected by single loss of *egl-13* and *ahr-1*, whereas loss of both genes totally abrogates expression, suggesting that *egl-13* and *ahr-1* act in parallel pathways to regulate *flp-8::gfp* expression (Figure S5). However, in the case of *flp-19::gfp*, loss of *egl-13* causes complete loss of expression and *ahr-1* plays a minor role in its regulation (Figure S5).

To further investigate the regulatory relationship between *egl-13*, *ets-5* and *ahr-1* we analyzed how they affect the expression of each other. We have already shown that *ets-5* positively regulates the expression of *egl-13* in the BAG neurons (Figure 5B). In a reciprocal experiment, we found that *ets-5::gfp* expression is unaffected in *egl-13(ku194)* mutant animals (Figure 5B). These data and other work [26], [33] suggest that *ets-5* acts upstream and in parallel to *egl-13* to direct BAG cell fate (Figure 5C). In addition, we found that *egl-13* is able to regulate its own expression in the BAG neurons, in parallel to *ets-5*; however, the mechanistic basis of this regulation is unclear (Figure 5B). In the URX neurons, we found that *egl-13* and *ahr-1* regulate the expression of each other in addition to having autoregulatory capabilities (Figure 5B,C and Figure S5).

Our studies have elucidated a novel function for *egl-13*, the SoxD homolog, in the specification of distinct classes of O_2_ and CO_2_ sensory neurons in *C. elegans*. We show that *egl-13* is expressed in the O_2_- and CO_2_-sensing neurons and acts cell-autonomously to regulate their distinct cell fates. We further show that *egl-13* is continuously expressed in the O_2_- and CO_2_-sensing system to maintain the expression of terminal features of these neurons. In certain cellular contexts, *egl-13* is also sufficient to induce O_2_- and CO_2_-sensing neuron cell fate. We found that the regulatory inputs controlling the expression of *egl-13* in the O_2_- and CO_2_-sensing system are mechanistically distinct. Independent regulatory modules control *egl-13* expression in the BAG neurons (CO_2_ and O_2_ downshift sensors) versus the URX neurons (O_2_ upshift sensors). Interestingly, we found that *egl-13* expression in the BAG neurons is controlled by the ETS-5 transcription factor via conserved ETS binding sites. In contrast, in the URX neurons, *egl-*13 expression is controlled by the bHLH transcription factor AHR-1 via a conserved AHR1 binding site.

The influence EGL-13 exerts on the expression of the terminal gene batteries of the distinct O_2_- and CO_2_-sensing neurons is diverse. Particular factors are exquisitely sensitive to loss of *egl-13*, whereas others are only partially affected. These findings suggest that alternative unknown modes of regulation are in place to ensure that particular molecules are faithfully expressed in the O_2_ and CO_2_ sensory neurons, which work in conjunction with and/or in parallel to *egl-13*.

Sox transcription factors have diverse functions during development and play crucial roles in regulating neuronal fate [34]–[37]. In addition, Sox proteins act at different levels to preselect neuronal genes in embryonic stem cells and to direct the activation of these genes in neuronal precursors and fully differentiated neurons [38]. Here we describe a novel role for EGL-13, the SoxD transcription factor in *C. elegans*, in driving the specification of different but related sensory neuron identities. Closely related orthologs of EGL-13 are found in vertebrates, some of which are expressed in sensory neurons [39], therefore; SoxD proteins may have a previously unrecognized conserved function in the specification of gas-sensing neurons in higher organisms.

## Materials and Methods

### Strains used in this study

Strains were grown using standard growth conditions on NGM agar at 20°C on *Escherichia coli* OP50 [8], [40]. Transgenic animals were created according to [41]. Strain information is detailed in Table S2.

### Forward genetic screening approaches

In all screens, animals were mutagenized with EMS (ethyl methanesulfonate) according to standard protocols [42]. Worms were incubated at 25°C at all times. In the manual screens, 5 parental (P0) mutagenized animals were placed in each of 10 founder plates. Three days later, 400 F1 progeny of the mutagenized P0 animals were singled. Their ensuing F2 progeny were screened under a fluorescence stereomicroscope.

In the automated worm sorter screen, around 100,000 synchronized larval stage L4 animals were mutagenized with EMS, the following day the P0 young adult animals were bleached and their F1 progeny synchronized at larval stage L1 by starvation (approximately 1,000,000 animals). F1 animals were grown to the young adult stage, bleached and their F2 progeny synchronized at larval stage L1 by starvation (approximately 10,000,000 animals). The F2 progeny were grown until larval stage L4 and 10% of the population (approximately 1,000,000) was passed through a COPAS biosorter (Karolinska Institute, Stockholm, Sweden).

### *C. elegans* expression constructs and generation of transgenic worms

Reporter gene constructs were generated by PCR amplifying promoter elements and cloning into the pPD95.75-mCherry and gfp vectors (Fire Vector Kit). Mutagenesis was performed using the QuikChange II XL Site-Directed Mutagenesis Kit (Stratagene). Rescue constructs were generated by cloning promoter and cDNA sequences into the pPD49.26 expression vector (Fire Vector Kit). Constructs were injected into young adult hermaphrodites as either simple arrays (*gcy-33^prom^::gcy-33::gfp* (50 ng ul^−1^) and pRF4 (50 ng ul^−1^) as injection markers) or as complex arrays using 1–10 ng ul^−1^ of linearized plasmid, 150 ng ul^−1^ of *PvuII*-digested bacterial genomic DNA and *myo-2^prom^::dsRed* (3–5 ng ul^−1^), *elt-2^prom^::gfp* (3–15 ng ul^−1^) as injection markers.

### Behavioral assays

Animals were transferred without food to 14 cm NGM assay plates containing a cut out arena of Whatman filter paper soaked in 20 mM CuCl_2_ to prevent them from leaving a 56 mm×56 mm center area. Sixty to seventy animals were used in a single experiment and starved for one hour prior to examination. Each experiment was carried out three times, except for wild-type, which was performed six times. A custom-made transparent plexiglass device with a flow arena of 60 mm×60 mm×0.7 mm was placed onto the assay arena and animals were accustomed to a gas flow of 100 ml/min containing 21% (v/v) oxygen for 5 minutes. During the assays animals were exposed for 6 minutes to 21% O_2_ before and after a 6 minute stimulus interval of either 10% O_2_ or 1% CO_2_ (+21% O_2_). All gas mixtures were balanced with N_2_. Gases were mixed with a static mixing element connected to mass flow controllers (Vögtlin Instruments) that were operated by LabView software. Recordings of freely behaving animals illuminated with flat red LED lights were made at 3 fps on a 4 megapixel CCD camera (Jai) using Streampix software (Norpix). Movies were analyzed by MatLab-based image processing and tracking scripts as previously described [43], [44]. The resulting trajectories were used to calculate instantaneous speed during continuous forward movements (1 second binning). Omega turns were detected based on characteristic changes in object eccentricity and their frequency was calculated in 15 second bins. For quantifications, relative speed changes were calculated between representative intervals of 120 seconds before (basal level) and 4 seconds after the stimulus, capturing the minimum speed levels (4–8 seconds post stimulus). Data were normalized to the basal level. Changes in omega turn frequency were calculated between representative intervals of 180 seconds before (basal level) and 60 seconds after the stimulus, to capture the maximum rise phase (55–115 seconds post stimulus).

### Heat-shock experiments

Two transgenic lines for *hsp-16.2::egl-13* were used for the heat-shock experiments. For the rescue and maintenance experiments, third larval stage (L3) worms were heat shocked at 37°C two times for 30 min. After heat shock, worms were kept at 25°C overnight and then transferred to 15°C for 2 days.

### Microscopy

Worms were mounted on 5% agarose on glass slides and images were taken using an automated fluorescence microscope (Zeiss, AXIO Imager M2) and MicroManager software (version 3.1).

### Neuronal scoring

Neurons were given a numerical value according to their expression levels. Wild-type expression scored 1, decreased expression scored 0.5 and abolished expression scored 0. Percentage of GFP expressing animals was then correlated to the theoretical maximum score using the equation below.
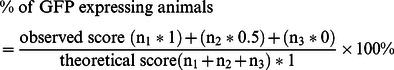



### Bioinformatic analysis

The Jaspar program (http://jaspar.genereg.net/) was used to predict the transcription factor binding sites in the *egl-13* upstream regulatory sequence.

### Statistical analysis

Statistical analysis was performed in GraphPad Prism 5 using one-way ANOVA with Newman-Keuls Multiple Comparison Test. Values are expressed as mean ± s.d. Differences with a *P* value<0.05 were considered significant. For the behavioral assays statistical significance was determined using one-way ANOVA with Bonferroni's Multiple Comparison Test.

## Supporting Information

Figure S1 *egl-13* mutants have anchor cell fusion defects. (A) *egl-13(ku194)* mutant hermaphrodites are severely egg-laying defective due to a defect in anchor cell fusion (right, compared to the wild-type adult on the left). (B) *egl-13* mutant alleles isolated from our forward genetic screens (*rp14*, *22*, *23* and *26*) all have anchor cell fusion defects comparable to the *ku194* allele. In the wild-type vulva, the anchor cell fuses to the utse cells to form the mature uterine-vulval connection (green arrowhead, top left). In *egl-13* mutant animals, the anchor cell fails to fuse to the utse (red arrowheads) and blocks egg-laying. (C) Scoring of neuronal phenotypes in *egl-13* mutants. *rp13* was isolated with the BAG marker, *gcy-33^prom^::gfp. rp22* and *rp23* were isolated with the URX marker, *flp-8^prom^::GFP* and *rp26* was isolated with the BAG, URX, AQR and PQR marker, *gcy-33^prom^::GCY-33::gfp*. Expression of each marker in wild-type animals is stated with a +. Scorings were conducted as stated in the materials and methods section.
(EPS)

Figure S2 BAG and URX lineages. Lineage diagrams of the BAG and URX neurons. Neurons whose fate are affected in *egl-13(ku194)* mutants are indicated in red, unaffected in green and untested in black. See Table S2 for fate markers used.
(EPS)

Figure S3 *egl-13* expression pattern. Expression pattern of *egl-13* at different stages (330–360 mins, 550 mins and L1 larva), using the *egl-13^prom1^::gfp* reporter transgene. Fluorescence micrographs (right) and differential interference contrast (DIC) microscopy images (left). Scale bar, 10 µm.
(EPS)

Figure S4 *egl-13* acts cell autonomously to control O_2_- and CO_2_-sensing neuron fate. (A) Protein domain structure of EGL-13 isoforms A+D. (B) Extrachromosomal transgenic rescue of *egl-13(ku194)* mutant phenotypes, in the *gcy-33^prom^::gcy-33::gfp* strain, using *egl-13^prom1^*-driven *egl-13^isoformA^cDNA* (left) and *egl-13^isoformD^cDNA* (right). Both isoforms equally rescue the *egl-13(ku194)* mutant phenotype. n = 46–61. *P<0.05, ***P<0.001 indicates significant difference from non-transgenic *egl-13* mutant animals. (C) Transgenic lines expressing *egl-13* cDNA under the control of *egl-13^prom1^* rescues all the *egl-13* alleles retrieved from the genetic screens. Rescue of *rp14* in BAG (*gcy-33^prom^::gfp), rp22* in URX (*flp-8^prom^::gfp*), *rp23* in URX (*flp-8^prom^::gfp*) and *rp26* in BAG, URX, AQR and PQR (*gcy-33^prom^::GCY-33::gfp*). n = 47–87. **P<0.01, ***P<0.001 indicates significant difference from non-transgenic *egl-13* mutant animals. See materials and methods for neuronal scoring criteria used.
(EPS)

Figure S5 Differential regulation of O_2_- and CO_2_-sensing neuron terminal differentiation markers by *egl-13*, *ets-5*, and *ahr-1*. (A) Requirement of *egl-13* and *ets-5* for the expression of BAG terminal differentiation markers: *flp-13*, *flp-17, flp-19*, *gcy-9, gcy-31* and *gcy-33. egl-13* and *ets-5* are both absolutely required for the expression of *flp-13* and *flp-19* suggesting that they act in a common pathway. *gcy-9* is predominantly regulated by *ets-5* with *egl-13* playing a lesser role. *flp-17* is regulated by both *egl-13* and *ets-5* acting in parallel pathways. Finally, *gcy-31* and *gcy-33* expression is predominantly regulated by *egl-13*. + = wt, red bars = mutant backgrounds. (B) Requirement of *egl-13* and *ahr-1* for the expression of URX terminal differentiation markers: *flp-8*, *flp-19* and *gcy-33*. *egl-13* and *ahr-1* act in parallel pathways to regulate *flp-8* expression. *egl-13* is absolutely required for the expression of *flp-19* whereas *ahr-1* is less important. *egl-13* and *ahr-1* are both required for *gcy-33* expression, probably via the same pathway. + = wt, blue bars = mutant backgrounds. Statistical model applied is a one-way ANOVA with Newman-Keuls multiple comparison test **P<0.05, ***P<0.005. Statistical model applied is a one-way ANOVA with Newman-Keuls multiple comparison test **P<0.05, ***P<0.005. Alleles used in this analysis *egl-13(ku194)*, *ets-5(tm1734)* and *ahr-1(ia03)*. See materials and methods for neuronal scoring criteria used.
(EPS)

Table S1 Expression of the O_2_- and CO_2_-sensing neuron terminal gene battery is affected in *egl-13* mutant animals. (A) Effects in the expression of O_2_- and CO_2_-sensing neuron reporters in *egl-13(ku194)* mutant animals differ from gene to gene and from cell to cell. For example, *flp-19* expression is strongly affected in both URX and BAG neurons, whereas *gcy-33* expression is more strongly affected in the URX neurons than BAG neurons. Fluorescent reporters for the sister cells of the URX (CEPD) and BAG (SMDV) neurons are unaffected in *egl-13(ku194)* mutant animals. (B) The deletion in the *egl-13* locus of *rp23* leaves the BAG-element intact (see Figure 5). As a result, BAG terminal fate marker expression is not significantly affected in *egl-13(rp23)* mutant animals. In both tables, quantification indicates the percentage of animals in which fluorescence was observed (ON), not observed (OFF) or asymmetrically affected left expressed (L ON) or right expressed (R ON). “−” indicates that the reporter is not expressed in those neurons. Animals were scored at the young adult stage. See materials and methods for neuronal scoring criteria used.
(EPS)

Table S2 Strains used in this study.
(EPS)
