# Correction: EGL-13/SoxD Specifies Distinct O_2_ and CO_2_ Sensory Neuron Fates in *Caenorhabditis elegans*

**DOI:** 10.1371/annotation/6f1c3fd1-c331-428c-9fae-513a2b11b2d9

**Published:** 2013-08-27

**Authors:** Jakob Gramstrup Petersen, Teresa Rojo Romanos, Vaida Juozaityte, Alba Redo Riveiro, Ingrid Hums, Lisa Traunmüller, Manuel Zimmer, Roger Pocock

There were errors in the funding section. The correct funding information is as follows:

This work was supported by a grant from the Lundbeck Foundation (www.lundbeckfonden.dk) (grant number R93-A8391), by an ERC Starting Grant (281869—elegansNeurocircuits), and by an ERC Starting Grant (260807 - HYPOXICMICRORNAS). The funders had no role in study design, data collection and analysis, decision to publish, or preparation of the manuscript.

